# Extensive amplification of GI-VII-6, a multidrug resistance genomic island of *Salmonella enterica* serovar Typhimurium, increases resistance to extended-spectrum cephalosporins

**DOI:** 10.3389/fmicb.2015.00078

**Published:** 2015-02-10

**Authors:** Ken-ichi Lee, Masahiro Kusumoto, Tsuyoshi Sekizuka, Makoto Kuroda, Ikuo Uchida, Taketoshi Iwata, Susumu Okamoto, Kimiko Yabe, Takashi Inaoka, Masato Akiba

**Affiliations:** ^1^Bacterial and Parasitic Disease Research Division, National Institute of Animal HealthIbaraki, Japan; ^2^Pathogen Genomics Center, National Institute of Infectious DiseasesTokyo, Japan; ^3^Hokkaido Research Station, National Institute of Animal HealthHokkaido, Japan; ^4^Food Biotechnology Division, National Food Research InstituteIbaraki, Japan; ^5^Graduate School of Life and Environmental Sciences, Osaka Prefecture UniversityOsaka, Japan

**Keywords:** resistance, genomic island, insertion sequence, gene duplication and amplification, *Salmonella enterica* serovar Typhimurium

## Abstract

GI-VII-6 is a chromosomally integrated multidrug resistance genomic island harbored by a specific clone of *Salmonella enterica* serovar Typhimurium (*S*.Typhimurium). It contains a gene encoding CMY-2 β-lactamase (*bla*_CMY−2_), and therefore contributes to extended-spectrum cephalosporin resistance. To elucidate the significance of GI-VII-6 on adaptive evolution, spontaneous mutants of *S*. Typhimurium strain L-3553 were selected on plates containing cefotaxime (CTX). The concentrations of CTX were higher than its minimum inhibition concentration to the parent strain. The mutants appeared on the plates containing 12.5 and 25 mg/L CTX at a frequency of 10^−6^ and 10^−8^, respectively. No colonies were observed at higher CTX concentrations. The copy number of *bla*_CMY−2_ increased up to 85 per genome in the mutants, while the parent strain contains one copy of that in the chromosome. This elevation was accompanied by increased amount of transcription. The *bla*_CMY−2_ copy number in the mutants drastically decreased in the absence of antimicrobial selection pressure. Southern hybridization analysis and short-read mapping indicated that the entire 125 kb GI-VII-6 or parts of it were tandemly amplified. GI-VII-6 amplification occurred at its original position, although it also transposed to other locations in the genome in some mutants, including an endogenous plasmid in some of the mutants, leading to the amplification of GI-VII-6 at different loci. Insertion sequences were observed at the junction of the amplified regions in the mutants, suggesting their significant roles in the transposition and amplification. Plasmid copy number in the selected mutants was 1.4 to 4.4 times higher than that of the parent strain. These data suggest that transposition and amplification of the *bla*_CMY−2_-containing region, along with the copy number variation of the plasmid, contributed to the extensive amplification of *bla*_CMY−2_ and increased resistance to CTX.

## Introduction

Non-typhoidal *Salmonella* (NTS) infection causes enterocolitis with diarrhea and/or invasive disease with bacteremia. Extended-spectrum cephalosporins (ESCs) or fluoroquinolones are commonly used as a first-line agent for the treatment of NTS infections, if susceptibilities to antimicrobials are unknown. In particular, ESCs are preferred, because fluoroquinolones are not recommended for use in children (Hohmann, 2001). Therefore, emergence of ESC-resistant NTS poses a threat to public health. Resistance to ESCs among *Salmonella enterica* serovars is attributed to the acquisition of Ambler class A extended-spectrum β-lactamases (ESBLs) and/or Ambler class C cephamycinases (AmpC β-lactamases). Of these, CMY-2, an AmpC β-lactamase encoded by the *bla*_CMY−2_ gene, is the most frequently detected enzyme among NTS (Michael et al., 2006).

**Figure 1:**
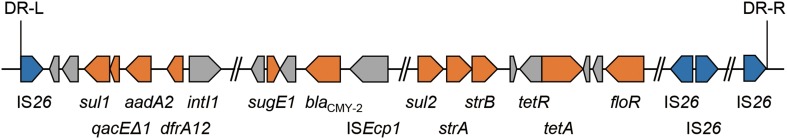
**Schematic view of antimicrobial resistance regions located in GI-VII-6**. Each arrow indicates gene according to the following scheme: blue, genes for IS*26* transposase; orange, resistance genes; gray, other genes. GI-VII-6 were flanked by an 8-bp direct repeat.

Human salmonellosis is mainly acquired by the ingestion of animal products, including meat, milk, and eggs (Mead et al., 1999). The use of antimicrobials in food-producing animals selects for bacteria resistant to antimicrobials used in humans (Phillips et al., 2004). We have continuously monitored the antimicrobial resistance of *S. enterica* serovar Typhimurium (*S*. Typhimurium) isolated from cattle in Japan (Tamada et al., 2001). Since 2004, *S*. Typhimurium isolates that are resistant to ESC have been detected in the northern major island, Hokkaido (Tamamura et al., 2011). Most of these ESC-resistant *S*. Typhimurium isolates have been noted to harbor one copy of *bla*_CMY−2_ in their chromosome as an element of a 125-kb genomic island, GI-VII-6 (Figure 1). A large proportion of GI-VII-6 (99%) shows a high sequence similarity (>99%) with *Escherichia coli* plasmid, pAR060302 (size, 167 kb). Directly repeated copies of insertion sequence IS*26* were present at both ends of GI-VII-6 (Shahada et al., 2011). Insertion sequences are the simplest transposable elements and can induce transposition of flanking genes to other replicons with themselves (Mahillon and Chandler, 1998). In fact, junction region of this genomic island were flanked by an 8-bp direct repeat, suggesting that GI-VII-6 was acquired by transposition involving IS*26* transposase (Shahada et al., 2011). Minimum inhibitory concentrations (MICs) of oxyimino-cephalosporins against these isolates have been found to be lower than those of other bacteria producing plasmid-encoded CMY-2 β-lactamases or ESBLs (Bonnet, 2004; Jacoby, 2009; Shahada et al., 2011). The significance of GI-VII-6 on ESC resistance and clonal spread of GI-VII-6 positive isolates is not yet clear.

Gene duplication and amplification (GDA) is known to be an important adaptive mechanism in both prokaryotes and eukaryotes under the existence of selection pressures such as anitimicrobials and carbon starvation (Andersson and Hughes, 2009). Bacterial genomic regions of up to 300 kb can be selected under antimicrobial selection pressures with the copy numbers of up to 40 (Sandegren and Andersson, 2009). The amplified genes alter the antimicrobial resistance phenotypes of the bacteria. For example, resistance of bacteria to sulfonamides, trimethoprim, and β-lactams can be increased by GDA of genes encoding antimicrobial hydrolytic enzymes, target enzymes, or efflux pumps (Andersson and Hughes, 2009; Sandegren and Andersson, 2009). Although the detailed mechanisms of GDA are still controversial, direct repeats flanking a region can contribute successful amplification (Sandegren and Andersson, 2009). The presence of directly repeated copies of IS*26* flanking GI-VII-6 (Shahada et al., 2011) prompted us to investigate whether increased ESC resistance due to GDA occurs in the *S*. Typhimurium isolates harboring GI-VII-6. The purpose of the present study is to understand the significance of GI-VII-6 in the adaptive evolution of *S*. Typhimurium under antimicrobial selection pressure.

Thus, we selected and analyzed spontaneous mutants of an *S*. Typhimurium strain harboring GI-VII-6 on cephalosporin-containing plates. GI-VII-6 was extensively amplified in all of the mutants by GDA mechanisms. However, the amplification pattern was more complex than we initially predicted. Pre-existing or transposed insertion sequences were observed at the junction of the amplified regions. The importance of insertion sequences on the adaptive evolution was highlighted in this study.

## Materials and methods

### Selection of *S*. typhimurium mutants with increased resistance to cefotaxime

To select *S*. Typhimurium spontaneous mutants with increased resistance to cefotaxime (CTX), L-3553 (Shahada et al., 2011) harboring GI-VII-6 in the chromosome was used as the parent strain. Whole-genome sequence of this strain was reported previously (Sekizuka et al., 2014). The MIC of CTX against this strain was 8 mg/L. This strain was inoculated into 3 ml of Luria–Bertani (LB) broth (Becton, Dickinson and Company, Franklin Lakes, NJ, USA) and incubated at 37°C and 150 rpm for 20 h. The shaking speed employed for broth culture was the same throughout this study. The culture was serially diluted and spread on LB agar plates (Becton, Dickinson and Company) containing 12.5, 25, 50, or 100 mg/L CTX (Sigma-Aldrich Co. LLC., St. Louis, MO, USA). In addition, the same culture was spread on LB agar plates without antimicrobials. The frequency of the mutations was estimated from the number of colonies on LB agar plates with CTX divided by that on plates without CTX. Twenty colonies were picked from the CTX-containing plates and inoculated into fresh LB broth with CTX (at the same concentration to that used in the initial selection) and incubated at 37°C for 24 h under constant shaking. Each culture was mixed with same volume of 50% (vol/vol) glycerol and stored at -80°C until further use.

### Determination of MIC

MIC was determined by the agar dilution method using Mueller–Hinton agar (Becton, Dickenson and Company), according to the procedure recommended by the Clinical and Laboratory Standards Institute (2008). The following internal quality control strains were used: *Staphylococcus aureus* ATCC 29213, *Enterococcus faecalis* ATCC 29212, *E. coli* ATCC 25922, and *Pseudomonas aeruginosa* ATCC 27853. The antimicrobials tested were as follows: CTX, ceftriaxone (CRO), ceftazidime (CAZ), cefoxitin (FOX), streptomycin (STR) (Sigma-Aldrich Co. LLC.), chloramphenicol (CHL) (Nacalai Tesque, Inc., Kyoto, Japan), and oxytetracycline (OTC) (Wako Pure Chemical Industries, Ltd., Osaka, Japan).

### Extraction of genomic DNA

Genomic DNA was extracted from 1 ml of 24-h culture using Wizard Genomic DNA Purification Kit (Promega Corporation, Madison, WI, USA), and used for quantitative real-time PCR (qPCR), PCR, and short-read DNA sequencing.

### qPCR

Primers used in this study for qPCR are listed in Table S1. Oligonucleotides were purchased from Hokkaido System Science Co., Ltd. (Sapporo, Japan). The qPCR technique was used for the quantification of relative copy number of *bla*_CMY−2_. Each reaction mixture contained 1X THUNDERBIRD SYBR qPCR Mix (Toyobo Co., Ltd., Osaka, Japan), 0.1X ROX (Toyobo Co., Ltd.), and 0.15 μM of each primer. The thermal conditions were initial denaturation at 95°C for 1 min, followed by 30 cycles of 95°C for 15 s and 60°C for 40 s. The signal was monitored using ABI Prism 7500 Real-time PCR System (Life technologies, Carlsbad, CA, USA). Standard curve method was used for relative quantification of target genes (Peirson et al., 2003). The *rpsJ* and *dnaN* genes were used as a single copy control in all experiments. The copy number was generated from a ratio of the relative amount of *bla*_CMY−2_ to that of a single copy gene. DNA of the parent strain, L-3553, was used to normalize the *bla*_CMY−2_ copy number of the mutants in every individual run.

### Periodical quantification of *bla_CMY−2_* copy number and mRNA expression

One hundred microliter of the frozen stock of each strain was inoculated into 100 ml of LB broth containing CTX and incubated at 37°C under constant shaking. The optical density at 600 nm (OD_600_) of each culture was measured and a portion of the culture was collected for DNA and RNA extraction in early logarithmic phase (OD_600_ = 0.5), middle logarithmic phase (OD_600_ = 1.5), late logarithmic phase (OD_600_ = 2.5), early stationary phase (OD_600_ = 3.0), and late stationary phase (24 h). The genomic DNA was extracted as described above and total RNA was extracted using RNAprotect Bacteria Reagent and RNeasy Mini Kit (Qiagen, Venlo, Netherlands). mRNA was converted to cDNA using the PrimeScript RT reagent Kit (Takara Bio Inc., Shiga, Japan) with random hexamers provided with the kit. qPCR was performed as described earlier to determine the relative expression level of mRNA for *bla*_CMY−2_. The parent strain, L-3553, was used to normalize the mRNA fold change. Because our preliminary data suggested that the *nrfG* expression was stable throughout different growth stages (data not shown), it was used as an internal control for mRNA quantification.

### PCR and DNA sequencing

Primers used in this study for PCR are listed in Table S1. PCR was performed using an iCycler apparatus (Bio-Rad Laboratories, Hercules, CA, USA), and Takara Ex Taq (Takara Bio Inc.) was used as the DNA polymerase for each PCR. The amplified fragments were purified using the ExoSAP-IT (USB Corporation, Cleveland, OH, USA), and the nucleotide sequence of both strands was determined using an Applied Biosystems 3130xl Genetic Analyzer with the BigDye Terminator cycle sequencing kit (version 3.1; Applied Biosystems, Foster City, CA, USA). The sequences were then assembled with Sequencher version 4 (Hitachi Solutions, Kanagawa, Japan) and the DNA alignment was examined using the Basic Local Alignment Search Tool (http://blast.ncbi.nlm.nih.gov/Blast.cgi).

### Short-read DNA sequencing and mapping on GI-VII-6 sequence

DNA libraries (insert size: ~600 bp) were prepared using the Nextera XT DNA Sample Prep Kit (Illumina, San Diego, WI). DNA cluster generation and paired-end sequencing run for 170 mers were performed using an Illumina MiSeq with the MiSeq 500 cycle Kit v2. The fluorescent images were analyzed using the Illumina RTA1.17.22/MCS2.1.43 base-calling pipeline to obtain FASTQ-formatted sequence data. To generate short-read mapping data of all strains compared to the chromosome and plasmid, pST3553 of *S*. Typhimurium L-3553 [DNA Data Bank of Japan (DDBJ) accession numbers AP014565 and AP014566, respectively], BWA-SW (Li and Durbin, 2010), and SAMtools (Li et al., 2009) software were used with the default parameters. The mapping data obtained were visualized with GenomeJack viewer software (Mitsubishi Space Software, Tokyo, Japan), and the mean coverage, which means average value of coverage of short reads on a specific genomic region, was calculated using R software version 3.0.0 (R Core Team, 2013).

### Southern hybridization analysis

To confirm the chromosomal location of *bla*_CMY−2_ and relevant genes among the strains L-3553 and its derivatives, pulsed-field gel electrophoresis (PFGE) followed by Southern hybridization analysis were performed according to methods described previously (Shahada et al., 2011), with some modifications. In brief, a plug of each strain was prepared using 24-h cultures. The plugs were digested with 60 U/ml of XbaI (Takara Bio Inc.) or 40 U/ml of FseI (New England BioLabs, Ipswich, MA, USA) at 37°C for 6 h, or 2 U/ml of S1 nuclease (Takara Bio Inc.) at 37°C for 45 min. Primers used for probe synthesis were listed in Table S1.

### Statistical analysis

Wilcoxon rank sum test was used to compare the *bla*_CMY−2_ copy number between increased CTX resistance mutants selected from the 12.5 and 25 mg/L CTX-containing plates. *R*^2^-value between the copy number of the genomic DNA and the mRNA fold change in *bla*_CMY−2_ in all strains tested was calculated. These analyses were performed using R software version 3.0.0.

### Nucleotide sequence accession numbers

The sequence reads obtained from the *S*. Typhimurium strains were deposited in DDBJ Sequence Read Archive under the accession number DRA001681.

## Results

### Frequency of selection and copy number of *bla*_CMY−2_ gene in spontaneous mutants

Spontaneous mutants were selected on the plates containing 12.5 and 25 mg/L CTX with a frequency of 1.1 × 10^−6^± 6.0 × 10^−7^ and 3.4 × 10^−8^ ± 1.6 × 10^−8^ (mean ± SD), respectively. No colonies appeared on the plates containing 50 and 100 mg/L CTX, suggesting that the frequency was less than 1.2 × 10^−10^. Twenty colonies were picked from each of the plates containing 12.5 and 25 mg/L CTX, and the copy number of *bla*_CMY−2_ was quantified. In the preliminary experiments, we determined the *bla*_CMY−2_ copy number of selected strains for three times and calculated the coefficient of variation (CV = standard deviation divided by mean). CV for the biological replicate was 0.153, whereas that for the technical replicate was 0.102. As shown in Table 1, all the mutants harbored multiple *bla*_CMY−2_ copies, ranging from 5.9 to 85.3, while the average *bla*_CMY−2_ copy number in 10 colonies of L-3553 on the LB agar plate was 1.01 ± 0.06. Higher variance in copy numbers was observed in mutants selected at 25 mg/L CTX (9.0–85.3) than in mutants selected at 12.5 mg/L CTX (5.9–25.7). Moreover, the average copy number in the mutants selected from plates containing 25 mg/L CTX (24.2 ± 20.6) was higher than that from plates containing 12.5 mg/L CTX (17.1 ± 5.1), although the difference was not statistically significant (*p* = 0.62).

**Table 1:** **Copy numbers of *bla*_CMY−2_ and MICs of various antimicrobials for *S*. Typhimurium L-3553 and the spontaneous mutants selected on CTX-containing plates**.

**Strain**	**CTX concentration for selection (mg/L)**	***bla*_CMY−2_ copy no**.	**MIC (mg/L)^a^**							**Approx. size of plasmid (kb)^b^**
			**CTX**	**CRO**	**CAZ**	**FOX**	**CHL**	**STR**	**OTC**	
L-3553	Not applicable	1.0	8	8	32	16	64	256	128	130
12-1	12.5	16.5	64	128	256	128	256	512	128	130
12-2	12.5	16.5	32	128	256	128	256	512	128	130
12-3	12.5	16.8	32	64	256	128	256	512	128	130
12-4	12.5	15.3	32	128	256	128	256	512	128	130
12-5	12.5	13.8	64	64	256	128	256	>512	128	130
12-6	12.5	15.8	32	128	256	128	256	>512	128	130
12-7	12.5	15.5	32	128	256	128	256	512	128	130
12-8	12.5	15.7	64	128	256	128	256	>512	128	130
12-9	12.5	17.4	64	64	256	128	256	>512	128	130
12-10	12.5	15.9	32	64	128	128	256	512	128	130
12-11	12.5	25.7	64	64	256	128	256	512	128	255, 390, 510, 640
12-12	12.5	24.5	64	128	256	128	256	512	128	255, 390, 510, 640
12-13	12.5	18.5	64	128	256	128	256	512	128	255, 390, 510, 640
12-14	12.5	25.7	64	128	256	128	256	512	128	255, 390, 510, 640
12-15	12.5	7.1	64	128	256	128	128	256	128	130, 255
12-16	12.5	14.9	32	64	256	128	256	512	128	130
12-17	12.5	21.7	64	128	256	128	256	512	128	255, 390, 510, 640
12-18	12.5	21.8	64	128	256	128	256	512	128	255, 390, 510, 640
12-19	12.5	5.9	64	128	256	128	256	512	128	130
12-20	12.5	17.3	32	64	256	128	256	>512	128	130
25-4	25	11.1	128	256	>512	128	256	>512	128	320, 430
25-5	25	15.4	128	256	>512	512	256	>512	256	130
25-6	25	85.3	64	128	512	256	256	512	128	510
25-7	25	17.1	128	256	>512	512	256	>512	256	130
25-8	25	67.4	64	128	512	256	256	512	128	440
25-9	25	18.8	128	256	>512	256	256	>512	256	130
25-10	25	12.9	128	256	512	256	256	>512	256	130
25-11	25	9.0	128	256	512	512	64	128	256	130
25-12	25	12.1	128	256	>512	512	256	>512	256	130
25-13	25	12.0	128	256	512	256	256	>512	128	130
25-14	25	15.9	64	128	512	128	256	>512	128	130
25-15	25	44.8	128	256	512	512	256	512	256	320, 390, 475, 560
25-16	25	13.1	128	256	>512	512	64	>512	128	130, 340, 440
25-17	25	34.1	128	128	512	128	256	>512	128	185, 250, 375, 495, 620
25-18	25	9.3	128	128	512	256	256	>512	256	130, 640
25-19	25	13.6	128	128	512	256	256	>512	128	130, 640
25-20	25	33.2	128	256	>512	512	256	>512	256	510, 570, 640
25-21	25	12.7	128	256	>512	256	256	>512	128	130
25-22	25	33.4	128	256	512	256	256	256	128	390, 475, 560, 640
25-23	25	12.8	128	256	>512	512	256	>512	128	130, 640

a *CTX, cefotaxime; CRO, ceftriaxone; CAZ, ceftazidime; FOX, cefoxitin; CHL, chloramphenicol; STR, streptomycin; OTC, oxytetracycline*.

b *Plasmid size was estimated by PFGE after S1 nuclease digestion*.

### MICs of relevant antimicrobials against the mutants

To confirm the phenotypic changes of the mutants, we determined the MICs of six additional antimicrobials whose resistance genes were located in GI-VII-6: resistance to CRO, CAZ, and FOX is conferred by *bla*_CMY−2_, CHL resistance is conferred by *floR*, STR resistance is conferred by *aadA2*, *strA*, and *strB*, and OTC resistance is conferred by *tetA* (Figure 1). As shown in Table 1, the MICs of CTX, CRO, and FOX were found to increase by four- to 32-fold. The MIC of CAZ against some of the mutants was over the detection limit. MIC_50_ and MIC_90_ of cephalosporin antimicrobials against the mutants selected from plates containing 25 mg/L CTX were higher than those from plates containing 12.5 mg/L CTX (Table S2). The MIC of chloramphenicol against the mutants increased four-fold, except for two strains showing the same value as that of the parent strain, whereas MIC of streptomycin against the mutants increased more than two-fold, but the values were over the detection limit in half of the strains. MIC of oxytetracycline against the mutants was comparable to that of the parent strain.

### Periodical quantification of *bla*_CMY−2_ copy number and mRNA expression

**Figure 2:**
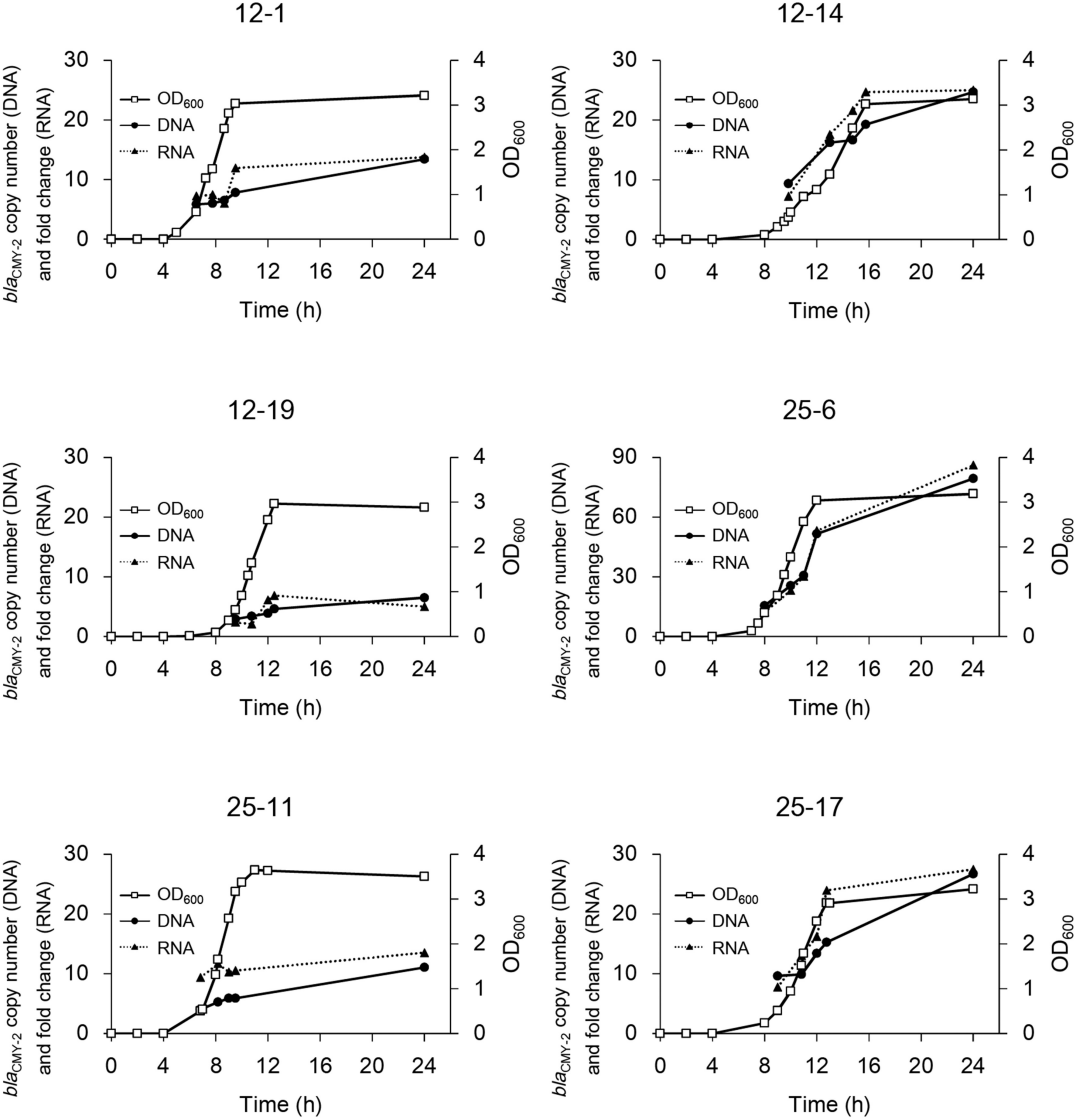
**Time courses of bacterial growth and *bla*_CMY−2_ gene amplification in the six selected mutants**. Bacterial growth is shown as OD_600_. The copy number of gene and fold change of mRNA are indicated. Note that the y-axis for strain 25-6 is different from that for the others.

*bla*_CMY−2_ copy number in the genomic DNA and fold change of mRNA were measured to confirm the correlation between them. As shown in Figure 2, the copy number of *bla*_CMY−2_ increased rapidly in the early logarithmic phase, and then gradually increased from late logarithmic to stationary phase in strains 12-1, 12-19, and 25-11. In contrast, the copy number increased linearly even in the stationary phase in strains 12-14, 25-6, and 25-17, leading to higher copy numbers. Furthermore, the fold changes of mRNA were significantly correlated with the *bla*_CMY−2_ copy numbers (*R*^2^ = 0.97, *p* < 10^−5^).

### Effect of serial subculture without CTX on *bla*_CMY−2_ copy numbers

**Figure 3:**
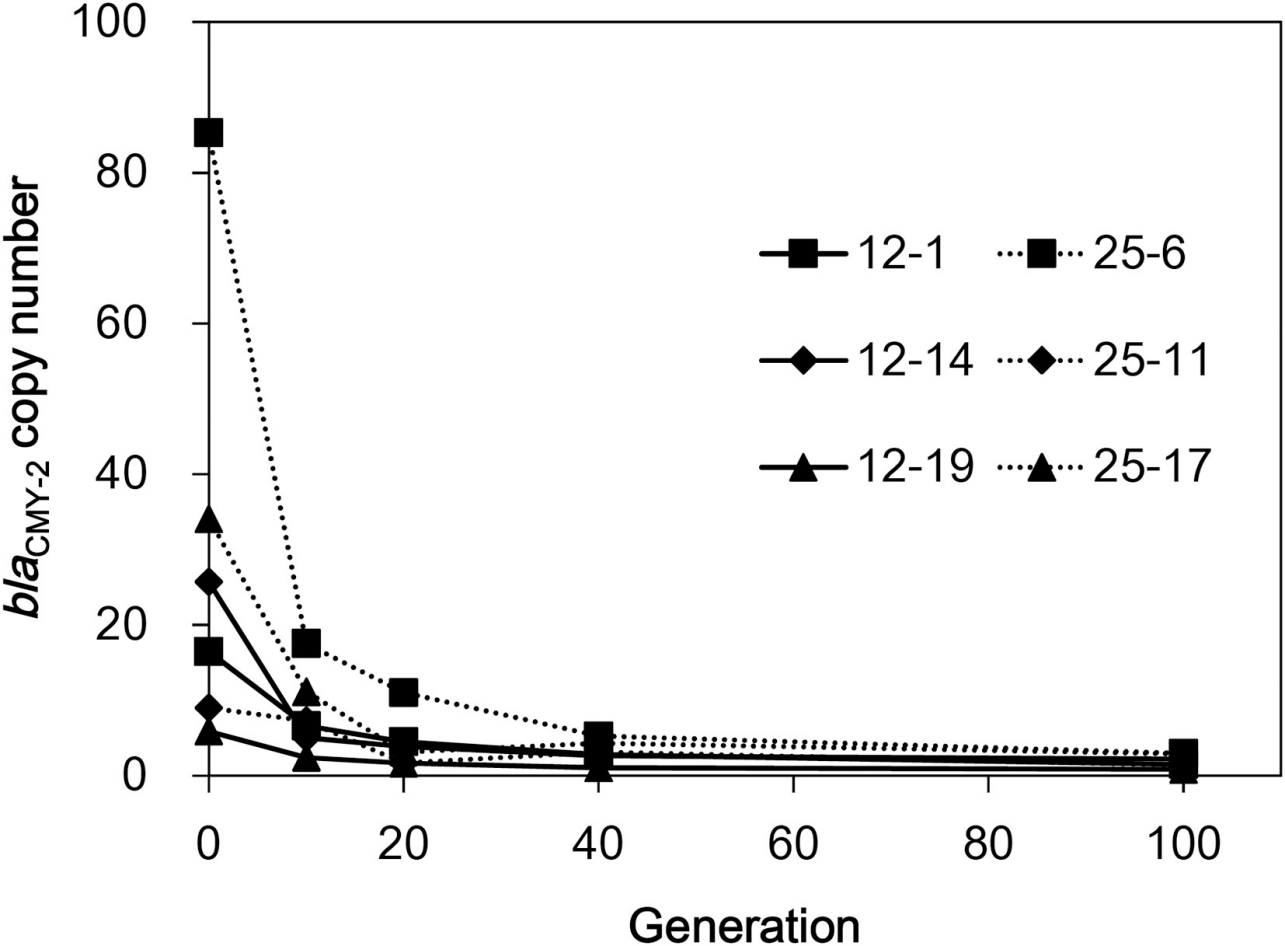
**The *bla*_CMY−2_ copy number reduction in the six selected mutants in the course of serial subculture without antimicrobials**.

To check the stability of the amplified regions in the absence of selection pressure, the six selected mutants were serially subcultured in LB broth without CTX for ten times (100 generations). As shown in Figure 3, the *bla*_CMY−2_ copy numbers drastically decreased in all isolates. Strain 25-6 showed approximately 80% decrease in the copy number in the first subculture. Subsequently, the copy number continuously decreased during the serial subculture, and finally reached a value of 3.0. The copy numbers of the other five strains ranged from 0.8 to 2.9 at 100 generations.

### Identification of mutations by short-read mapping

In the six selected mutants, no mutations were found in *bla*_CMY−2_, or its promoter region. Furthermore, no point mutations were observed among the mutants' whole-genome sequences, except for three non-synonymous mutations listed in Table S4. We observed a 51-kb deletion [nucleotide (nt) position 1763615–1814713] of chromosomal DNA in strain 25-17, although the XbaI and FseI restriction sites were not affected by these mutations.

### Localization of *bla*_CMY−2_ by southern hybridization analysis

**Figure 4:**
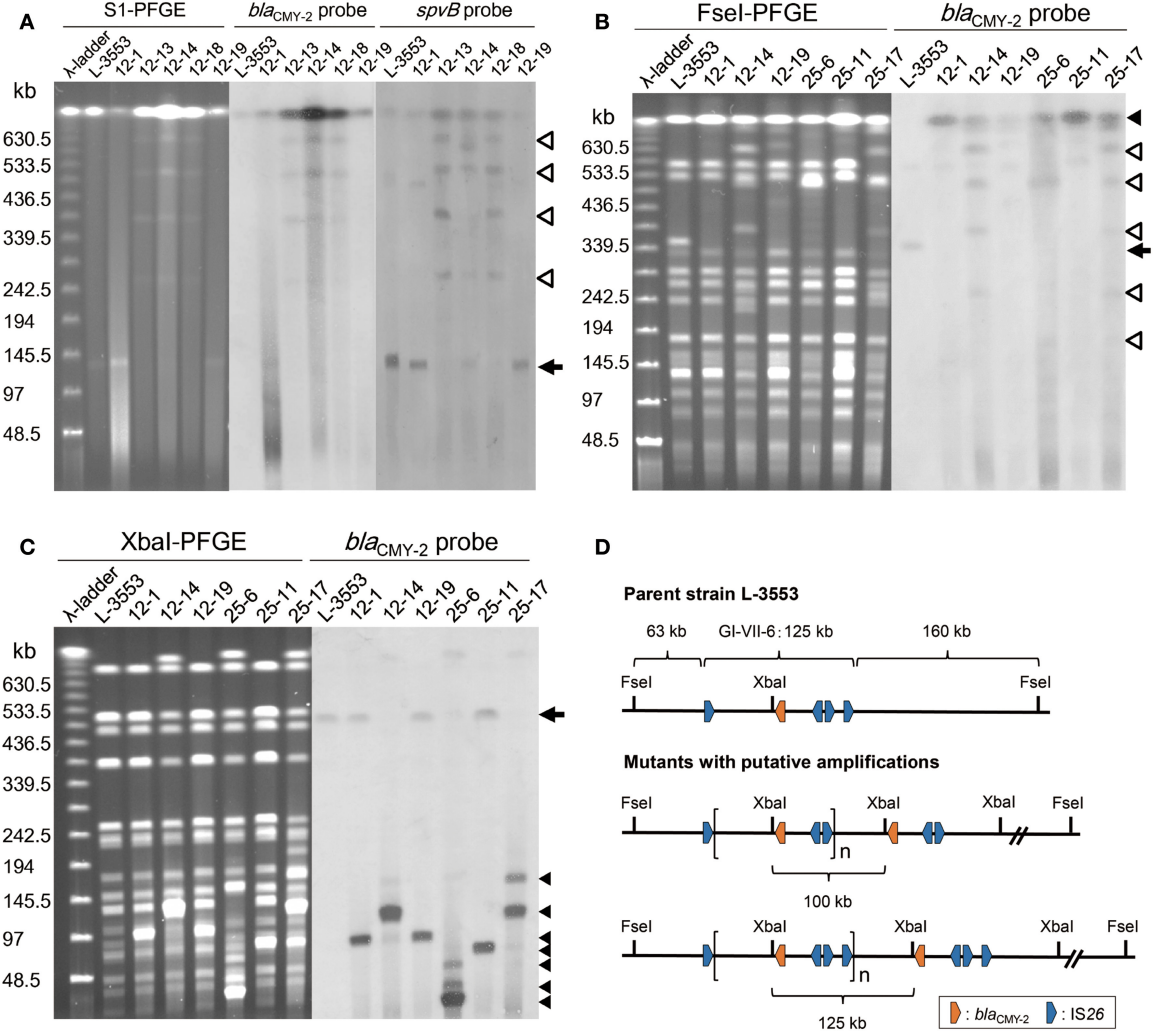
**PFGE–Southern hybridization images demonstrating *bla*_CMY−2_ and *spvB* locations in *S*. Typhimurium strains. (A)** PFGE separation of S1 nuclease-digested genomic DNA from the selected strains followed by Southern hybridization with probes specific to *bla*_CMY−2_ and *spvB*. An arrow indicates the position of the endogenous 133-kb plasmid, in which the *spvB* signal was detected. Open triangles indicate the positions of enlarged plasmids, in which both *bla*_CMY−2_ and *spvB* signals were detected. Both signals were observed in multiple plasmid bands in strains 12-13, 12-14, and 12-18. **(B)** PFGE separation of FseI-digested genomic DNA from the selected strains followed by Southern hybridization with a *bla*_CMY−2_ probe. An arrow indicates the original position of the restricted fragment (350 kb) in which the chromosomal *bla*_CMY−2_ signal was detected. In the selected mutants, this fragment was not present and the *bla*_CMY−2_ signal was identified on the largest band as indicated by a closed trangle. Open triangles indicate the position of enlarged plasmid observed in strains 12-14, 25-6, and 25-17. **(C)** PFGE separation of XbaI-digested genomic DNA from the selected strains followed by Southern hybridization with a *bla*_CMY−2_ probe. An arrow indicates the original position of the restricted fragment (520 kb) in which chromosomal *bla*_CMY−2_ signal was detected. Closed triangles indicate the position of *bla*_CMY−2_ signals that originated from tandemly amplified GI-VII-6. **(D)** Restriction maps of the GI-VII-6 and its flanking region in the parent strain L-3553 and the mutants with putative amplifications. One putative amplified region presented is flanked by the first and the third copies of IS*26* (upper), and the other is flanked by the first and the fourth copies of IS*26* (lower). These amplified regions generate 100-kb (upper) and 125-kb (lower) fragments after XbaI digestion, respectively.

The parent strain, L-3553, harbors a 133-kb plasmid, pST3553, which is a derivative of *S*. Typhimurium specific virulence plasmid and does not contain *bla*_CMY−2_ gene (Sekizuka et al., 2014). In some of the mutants, one or multiple plasmids with sizes larger than the original were observed. Estimation by S1 nuclease-PFGE indicated that the size of the plasmids varied from 255 to 660 kb (Table 1). Southern hybridization analysis revealed that *bla*_CMY−2_ and the virulence plasmid specific gene, *spvB*, were both present in the larger plasmids. In all strains tested, *bla*_CMY−2_ signal was also present in the largest band, which is originated from the chromosome (Figure 4A). *spvB* signals in the largest band seemed a non-specific one, because we observed the signal even in the parent strain L-3553.

To demonstrate the amplification of *bla*_CMY−2_ at the original chromosomal position, we performed additional PFGE–Southern hybridization analyses with a *bla*_CMY−2_ probe using FseI-digested genomic DNA of the six selected mutants. As GI-VII-6 does not contain an FseI site, it was located in its entirety in a 350-kb band in L-3553 (Figures 4B,D). This band was not present in the genomic DNA extracted from the mutants. A *bla*_CMY−2_ probe was found to hybridize with the largest band originating from the chromosome in the selected mutants. Additional *bla*_CMY−2_ signals originating from enlarged plasmids were observed in strains 12-14, 25-6, and 25-17. As the endogenous pST3553 plasmid contains one FseI site, the sizes of the *bla*_CMY−2_-positive bands corresponded to those of the enlarged plasmids.

Because GI-VII-6 contains one XbaI site (nt 1037120–1037125) (Shahada et al., 2011), tandemly arrayed GI-VII-6 sequences should generate multiple copies of a specific sized fragment after XbaI digestion (Figure 4D). As shown in Figure 4C, we observed several bands of different sizes, which were not found in the parent strain, after separation of XbaI-digested genomic DNA of the selected mutants by PFGE. Most of them were approximately 30–125 kb in size and showed higher fluorescence intensity compared to the other bands. In subsequent Southern hybridization analysis, *bla*_CMY−2_ signal was observed in these bands. The sizes of the bands that showed the prominent *bla*_CMY−2_ signal were approximately 100, 125, 100, 30, 80, and 110 kb in strains 12-1, 12-14, 12-19, 25-6, 25-11, and 25-17, respectively. In strain 25-6, additional weaker signals were observed in the bands of approximately 40 and 85 kb in size. An additional weaker signal was also observed in a 180-kb band in strain 25-17.

### Estimation of plasmid copy number by short-read mapping

To estimate the plasmid copy number in the parent and each mutant, the mean coverage of the pST3553 plasmid was divided by that of the chromosome. The plasmid copy numbers of strains L-3553, 12-1, 12-14, 12-19, 25-6, 25-11, and 25-17 were estimated as 1.6, 2.3, 5.2, 2.7, 4.7, 1.9, and 7.1, respectively. Three strains with a *bla*_CMY−2_ copy number greater than 25 (12-14, 25-6, and 25-17) showed higher plasmid copy numbers.

### Identification of the amplified region by short-read mapping

**Figure 5:**
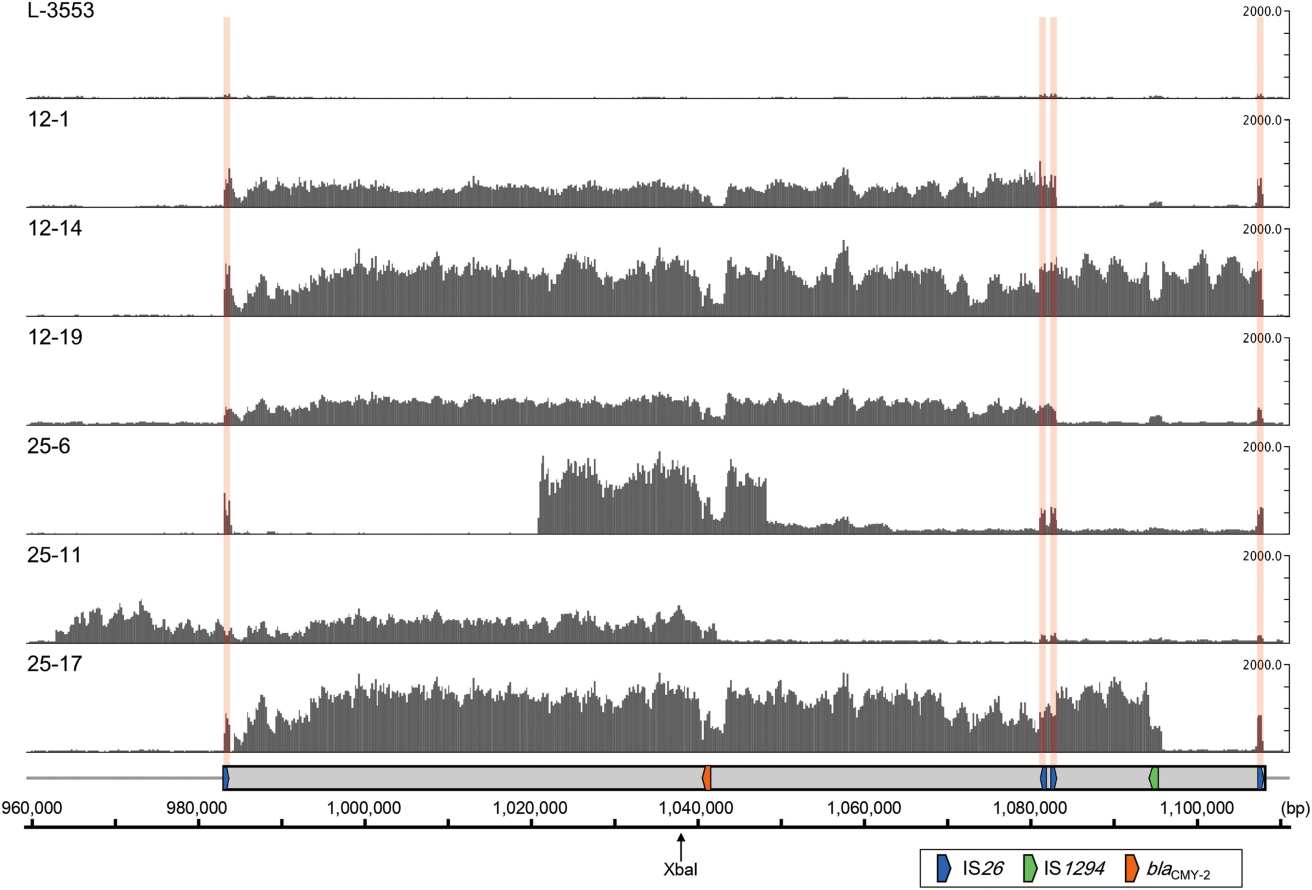
**Coverage of short-reads obtained from *S*. Typhimurium L-3553 and the six mutants (DDBJ Sequence Read Archive; accession number DRA001681) on the GI-VII-6 sequence of strain L-3553 (DDBJ accession number AP014565)**. In each row, the y-axis shows the coverage of the short-reads. The ruler at the bottom indicates the nucleotide number in the chromosome of strain L-3553. The XbaI restriction site is indicated by a slim arrow. The gray rectangle indicates the location of GI-VII-6. The colored arrows indicate the location of key genes as explained in the open rectangle. The locations of IS*26* are indicated by pink vertical bands. Short reads originated from IS*26* are mapped to one of four copies of IS*26* in GI-VII-6 randomly.

The results of the short-read mapping showed that the mean coverage of the chromosomal genes (background) in the six selected mutants ranged from 20.7 to 61.1 (Table S3). In this study, regions showing a mean coverage that is greater than five times higher than the background were regarded as amplified regions. As shown in Figure 5, coverage of the entire or a part of GI-VII-6 was higher than other regions in the six selected mutants. The amplified regions of strains 12-1, 12-14, and 12-19 were flanked by directly repeated copies of IS*26*. The end points of the amplified regions in strains 12-1 and 12-19 were the first and third copies of IS*26* (nt positions 983123–983942 and 1082604–1083423, respectively) from the 5′-end of GI-VII-6. The size of the amplified regions was 100 kb. In strain 12-14, the end point of the amplified region was the first and fourth (nt position 1107425–1108244) copies of IS*26*.

In contrast, we could not identify directly repeated sequences at the ends of the amplified regions in strains 25-6, 25-11, and 25-17. The amplified regions in strain 25-6 started from an intergenic region between STL3553_c09800 and STL3553_c09810 (nt position 1021052) and terminated at an intergenic region between STL3553_c10000 and STL3553_c10010 (nt position 1048473). The mean coverage of this amplified region was 1296.9. Furthermore, in this strain, the mean coverage from downstream of the amplified region to nt position 1063378, located in the middle of gene STL3553_c10130, was 231.1, which subsequently decreased to 102.5 until just before the fourth copy of IS*26*. These data suggest the existence of three different amplified regions [nt positions 1021052–1048473 (27 kb), 1021052–1063378 (42 kb), and 1021052–1108244 (87 kb)] sharing a portion of GI-VII-6 sequence. In strain 25-11, the 5′-end of the amplified region started from 21 kb upstream of the 5′-end of GI-VII-6 (nt position 962959) and terminated at the middle of IS*Ecp1* (nt position 1042417) within GI-VII-6, which accounted for a size of 80 kb. The coverage dropped sharply in the middle of IS*Ecp1*, while it was still higher by several times than the background until the 5′-end of the IS*Ecp1* inverted repeat (nt position 1043519) (Figure S1). In strain 25-17, high mean coverage of 1269.7 was observed from an intergenic region between STL3553_c09480 and STL3553_c09490 (nt position 984235) to the 3′-end of IS*1294* (nt position 1096148), which accounted for a size of 112 kb. (Figure S1). The sizes of these amplified regions in each mutant corresponded to those of the bands that showed the prominent or weaker *bla*_CMY−2_ signals (Figure 4C). Among the *bla*_CMY−2_ signals observed in Figure 4C, the origin of the 180-kb signal was not identified in this study.

### DNA sequence of the junction region

To determine the DNA sequence of the junction of the amplified region, we performed DNA sequencing of the PCR products. Location of each primer to amplify the junction region of strains 12-1, 12-14, 12-19, 25-6, and 25-11 is indicated in Figure S2. In strains 12-1, 12-14, and 12-19, the location of IS*26* at the junction was confirmed. In addition, we confirmed the location of IS*26* at the junctions of three different amplified regions in strain 25-6. The location of IS*1* at the junction of amplified region was also confirmed in strain 25-11 (see Figure S3). We could not successfully amplify the junction region in strain 25-17.

## Discussion

The present study demonstrates that increased cephalosporin resistance of *S*. Typhimurium harboring GI-VII-6 under CTX selection pressure is mainly based on the increased copy number of *bla*_CMY−2_ gene by GDA mechanisms. This conclusion is elicited from the following evidence. First, the mean coverage of the entire or a part of GI-VII-6 was greater than five times higher than that of other chromosomal regions (Table S3). Second, *bla*_CMY−2_-positive fragments that were 30–125 kb in size were observed after XbaI digestion of the genomic DNA (Figure 4C), suggesting a tandem array of the amplified regions. In addition, a *bla*_CMY−2_-positive chromosomal fragment of 350 kb in size was lost and a fragment of larger size appeared after FseI digestion by Southern hybridization analysis (Figure 4B), indicating that amplification occurred at the original position. Third, increased copy number of *bla*_CMY−2_ was accompanied by increased amount of transcription (Figure 2). Fourth, the *bla*_CMY−2_ copy number drastically decreased in the course of serial subculture without selection pressure (Figure 3). Lastly, the frequency of the spontaneous-mutants selection was 10^−6^ and 10^−8^ on 12.5 and 25 mg/L CTX-containing plates, respectively. These frequencies are similar to those reported in previous GDA studies and appear to be higher than those of point mutations. Above-mentioned characteristics are in concordance with those of GDA mechanisms reported previously (Andersson and Hughes, 2009; Sandegren and Andersson, 2009).

Two major mechanisms have been proposed that can increase gene copy number. One is the homologous and/or non-homologous recombination based replication and the other is rolling circle replication (Andersson and Hughes, 2009; Sandegren and Andersson, 2009). The initial step of the former mechanism is duplication either through non-equal homologous recombination between directly oriented repeats or through RecA-independent mechanisms between short- or no-homology regions. Following duplication, higher level amplification can occur as a result of RecA-dependent recombination between the tandem repeats generated by the initial duplication. Alternatively, no duplication intermediates are required in the rolling circle replication; a double-strand break allows single-strand invasion at a homologous or micro-homologous region, followed by amplification. Compared to non-equal homologous recombination, rolling circle replication can generate a large tandem array in a single generation (Sandegren and Andersson, 2009).

In the present study, the amplified regions in the three mutants selected on 12.5 mg/L CTX-containing plates had directly oriented IS*26* copies at both ends (Figure 5). The initial duplication appeared to have occurred between pre-existing IS*26* copies in these strains. In contrast, we could not find any homologous sequences at both ends of the amplified regions in the remaining three strains selected on 25 mg/L CTX-containing plates by short-read mapping. However, results of our PCR and sequencing analyses suggested that IS*26* was found at the junctions of the three different amplified regions in strain 25-6. Also, IS*1* was found at the junction of the amplified region in strain 25-11 (Figure S3). The IS*26* and/or IS*1* insertions at both ends of the amplified regions may have preceded the amplification in these mutants. In strain 25-6, we observed three prominent *bla*_CMY−2_ signals in Xba I-restricted fragments less than 100 kb in size (Figure 4C), suggesting these regions were amplified at different loci in the genome. Transposition of the *bla*_CMY−2_-containing region flanked by directly repeated IS*26* copies may have preceded the amplification in this mutant. This signifies that the amplification site is not exclusively in the original position. Because the parent strain contains four copies each of the IS*26* and IS*1* transposases (Sekizuka et al., 2014), these insertion sequences may transpose in the parent strain with a relatively high frequency. Reams et al. (2012) reported that 97% of duplication events resulted from recombination between IS*3* among non-selectively trapped 1800 spontaneous *lac* duplications, and that the formation of the duplications was stimulated by IS*3* transposase and plasmid transfer functions (TraI). As GI-VII-6 contains *traI*, the multiple copies of IS*26*, IS*1*, and *traI* may increase the duplication frequency mediated by IS*26* and IS*1*.

*S*. Typhimurium L-3553 harbors a 133-kb virulence and antimicrobial resistance plasmid, pST3553. We observed several plasmids larger than pST3553 in part of the spontaneous mutants. For example, the same set of four differently sized plasmids were detected in strains 12-11, 12-12, 12-13, 12-14, 12-17, and 12-18 by PFGE after S1 nuclease digestion with a size interval of 120–135 kb. We observed both *bla*_CMY−2_ and *spvB* signals in most plasmids (Figure 2), suggesting that these plasmids were cointegrates of the endogenous plasmids with different copies of GI-VII-6. We obtained *E. coli* transformant harboring the plasmid from strain 12-18. Results of PCR scanning to check the structure of GI-VII-6 (Shahada et al., 2011) revealed that the enlarged plasmid contained the entire GI-VII-6 sequence (data not shown). We thus concluded that the enlarged plasmids were generated by the transposition of GI-VII-6 into pST3553. The size of the plasmid is 38 times smaller than that of the chromosome. However, pST3553 shares several homologous regions with GI-VII-6 (Sekizuka et al., 2014). The regions would increase the frequency of integration of the GI-VII-6 into the plasmid mediated by homologous recombination. In addition, the plasmid copy number in the six selected strains was 1.4 to 4.4 times higher than that of the parent strain. Especially, strains with higher copy numbers of *bla*_CMY−2_ (12-14, 25-6, and 25-17) harbored enlarged plasmids and expressed higher copy numbers of plasmid. With the exception of the three non-synonymous mutations (Table S4), we could not detect any mutations among the genomic DNA of the selected mutants. Thus, these results may be due to the copy-number variation of the plasmid and selection of cells with higher copy-number of the plasmid under the selection pressure. These data suggest that transposition and amplification of the *bla*_CMY−2_-containing region, along with the copy number variation of the enlarged plasmid, contributed to the extensive amplification of *bla*_CMY−2_.

We showed that spontaneous mutants can be selected at CTX concentration marginally above the MIC. No mutants were detected on 50 and 100 mg/L CTX-containing plates, suggesting that GDA is amenable to the mutant selection window (MSW) theory (Drlica and Zhao, 2007). The MSW theory postulates that antimicrobial-resistant mutant subpopulations present before the initiation of antimicrobial treatment are enriched and amplified during the therapy when the antimicrobial concentrations remain in a specific range, the MSW. The upper boundary of the MSW is the minimum concentration that can inhibit the growth of all types of single mutants, called mutant prevention concentration (MPC). As duplication of any specific bacterial gene occurs at a high rate (10^−3^–10^−5^/cell/division) (Anderson and Roth, 1981; Reams et al., 2010), overnight culture of the parent strain should comprise a certain number of mutants with duplications mediated by IS*26* or IS*1*. These mutants may be enriched in the presence of CTX after amplification of the *bla*_CMY−2_-containing region with directly repeated copies of IS*26* or IS*1* at both ends. Although effective amplification may contribute to resistance against higher concentrations of CTX, there must be a limit, i.e., an increase in the genome poses a fitness cost to the host (Nilsson et al., 2006; Reams et al., 2010). In fact, we observed a drastic reduction in the *bla*_CMY−2_ copy number in the course of serial subculture without selection pressure.

Taken together, we conclude that GI-VII-6 amplification occurred at the original position, while it also transposed to other genomic locations, including an endogenous plasmid in some of the mutants. Insertion sequences played important roles in the transposition and amplification of *bla*_CMY−2_ containing regions. The copy-number variation in the *bla*_CMY−2_-positive plasmid partially contributed to the extensive amplification of *bla*_CMY−2_ in some of the mutants. In addition, the selection frequency of mutants with gene amplification was higher than that of typical mutations, suggesting that gene amplification is one of the adaptive mechanisms that occur prior to acquiring additional mutations in order to facilitate antimicrobial resistance.

### Conflict of interest statement

The authors declare that the research was conducted in the absence of any commercial or financial relationships that could be construed as a potential conflict of interest.
