# Supplementary material for: Extensive amplification of GI-VII-6, a multidrug resistance genomic island of Salmonella enterica serovar Typhimurium, increases resistance to extended-spectrum cephalosporins
Source: Front Microbiol. 2015 Feb 10;6:78. doi: 10.3389/fmicb.2015.00078 (PMC4322709; doi:10.3389/fmicb.2015.00078)
Supplement: Supplementary file 1 [file Table1.PDF]

TABLE S1. Primers used in this study

Target	Primer	Sequence (5'→3')	Product size (bp)
PCR			
rpsJ	ST-rpsJ-F2	TCCGTATCCGCCTGAAAG	151
	ST-rpsJ-R2	TGCGGAGAGATCAGAACG	
dnaN	ST-dnaN-F	TCTTTACCCAGCCACTCG	154
	ST-dnaN-R	CCACCAGCTTCGAGGTAA	
bla $_{CMY-2}$	qCMY2-F	GCTGCTGACAGCCTCTTT	197
	qCMY2-R	GCGTGACTGGGTGGTTAT	
nrfG	qnrfG-F	AGGCCGGGCAACATATGA	150
	qnrfG-R	AGGTTTGCCACAGCGACA	
right junction in 12-1 and 12-19	12-1 F2	CAGAAACGATTGACGCAGGC	1132
right junction in 12-14	12-14 F1	AGCTTTGGCCGCGTTGATGC	1076
left junction in 12-1, 12-14, and 12-19	12-1 R1	TCTGCCGAAGGTTGAAGGTC	
right junction A in 25-6	25-6 F1	GATGAATGAACCAGATGAGC	1092
right junction B in 25-6	25-6 F2	CCTGAGTATCCTCTTCTGTC	1058
right junction C in 25-6	25-6 F3	CAATCCTGGAAGGGGAGAAC	1072
left junction in 25-6	25-6 R1	TTCAAACCCATCGGGGCAAC	
left junction in 25-11	25-11 R1	ACCAGCCTTCATAATATCCCGCCAG	2146
Probe synthesis for Southern hybridization analysis			
bla $_{CMY-2}$	CMY-2-F	ATGATGAAAAAATCGTTATGCT	571
	CMY-2-R	CGTAACTCATTCCTGAGGGTT	
spvB	spvBF	AGCAGTTTTTATCGCCTGGA	526
	spvBR	GGTGGAACATCAGGACTTGG	
