# Supplementary material for: Extensive amplification of GI-VII-6, a multidrug resistance genomic island of Salmonella enterica serovar Typhimurium, increases resistance to extended-spectrum cephalosporins
Source: Front Microbiol. 2015 Feb 10;6:78. doi: 10.3389/fmicb.2015.00078 (PMC4322709; doi:10.3389/fmicb.2015.00078)
Supplement: Supplementary file 2 [file Table2.PDF]

TABLE S2. MIC_{50} and MIC_{90} of mutants selected using different concentrations of CTX

Indicator	CTX selection mg/L[a]	Antimicrobials[b]						
		CTX	CRO	CAZ	CFX	CHL	STR	OTC
MIC_{50}	12.5	64	128	256	128	256	512	128
	25	128	256	512	256	256	>512	128
MIC_{90}	12.5	64	128	256	128	256	>512	128
	25	128	256	>512	512	256	>512	256

[a]CTX concentration used for spontaneous mutant selection.

[b]CTX, cefotaxime; CRO, ceftriaxone; CAZ, ceftazidime; CFX, cefoxitin; CHL, chloramphenicol; STR, streptomycin; OTC, oxytetracycline.
