# Supplementary material for: Extensive amplification of GI-VII-6, a multidrug resistance genomic island of Salmonella enterica serovar Typhimurium, increases resistance to extended-spectrum cephalosporins
Source: Front Microbiol. 2015 Feb 10;6:78. doi: 10.3389/fmicb.2015.00078 (PMC4322709; doi:10.3389/fmicb.2015.00078)
Supplement: Supplementary file 3 [file Table3.PDF]

TABLE S3. Size and coverage of amplified regions in *S*. Typhimurium strains

Strain	Nucleotide position of amplified region	Size (bp)	Coverage (mean)	
			Amplified region[a]	Background[b]
L-3553	983123-1108244	125122	24.6	33.6
12-1	983123-1083423	100301	480.2	39.7
12-14	983123-1108244	125122	998.6	21.4
12-19	983123-1083423	100301	537.6	61.1
25-6	1021052-1048473	27422	1296.9	20.7
	1048474-1063378	14905	231.1	
	1063379-1108244	44866	102.5	
25-11	962959-1042417	79459	491.1	49.4
25-17	984235-1096148	111914	1269.7	36.2

[a]Except for IS*26* and IS*1294* located at both ends.

[b]Mean coverage against chromosome (DDBJ accession number AP014565).
