# Supplementary material for: Extensive amplification of GI-VII-6, a multidrug resistance genomic island of Salmonella enterica serovar Typhimurium, increases resistance to extended-spectrum cephalosporins
Source: Front Microbiol. 2015 Feb 10;6:78. doi: 10.3389/fmicb.2015.00078 (PMC4322709; doi:10.3389/fmicb.2015.00078)
Supplement: Supplementary file 4 [file Table4.PDF]

TABLE S4. Point mutations detected in the spontaneous mutants

Strain	Gene	Function	Position[a]		Substitution	
					Nucleotide	Amino acid
12-19		DNA polymerase III subunit protein	Chromosome	303,930	T→G	V→G
12-19	*samA*	Mutagenesis by UV and mutagens	Plasmid	83,397	T→G	F→V
25-6	*dcp*	dipeptidyl carboxypeptidase II	Chromosome	1,714,794	C→G	S→OPA

[a] Nucleotide position corresponds to the genome sequence of the parental strain (DDBJ accession number AP014565 and AP014566)
