# Supplementary material for: Extensive amplification of GI-VII-6, a multidrug resistance genomic island of Salmonella enterica serovar Typhimurium, increases resistance to extended-spectrum cephalosporins
Source: Front Microbiol. 2015 Feb 10;6:78. doi: 10.3389/fmicb.2015.00078 (PMC4322709; doi:10.3389/fmicb.2015.00078)
Supplement: Supplementary file 5 [file Image1.PDF]

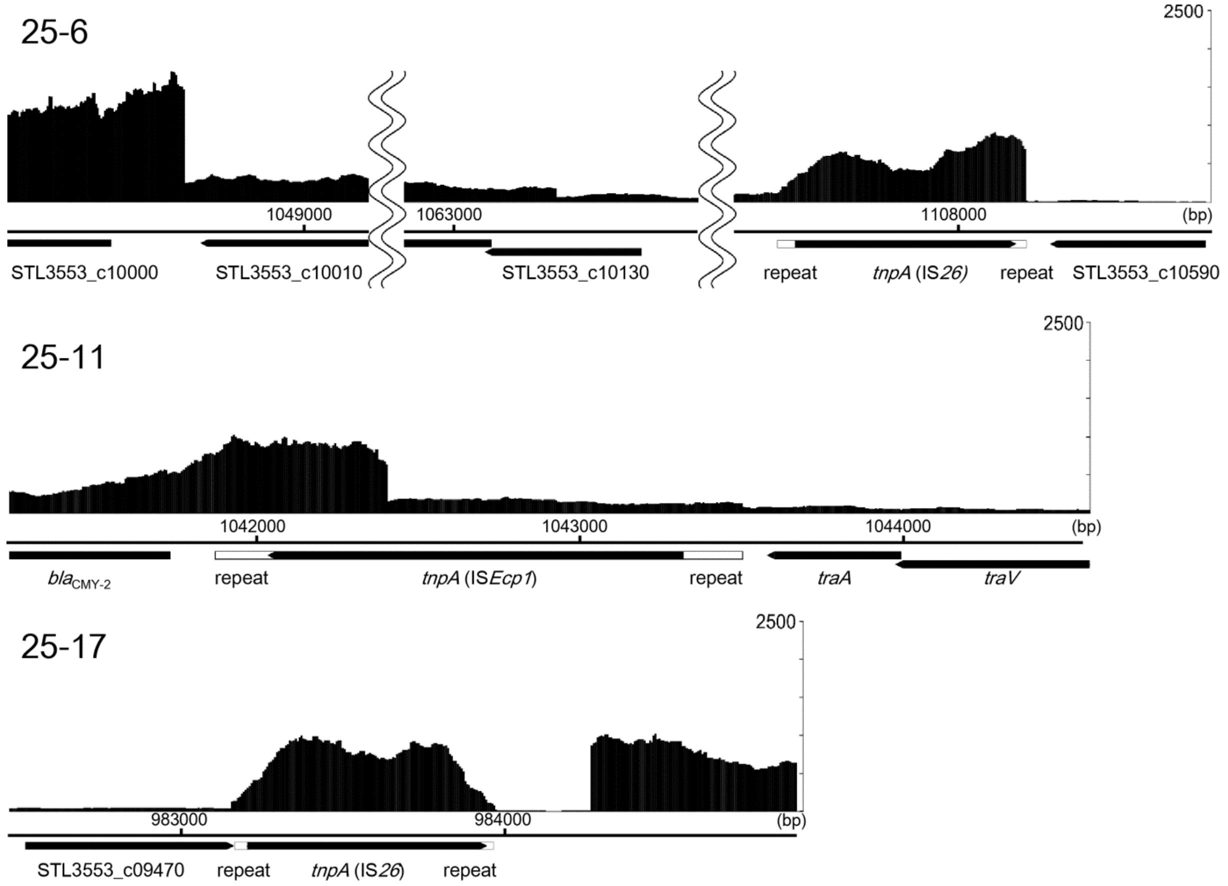

FIGURE S1. Magnified view of the boundary region of short-read coverage obtained from three mutants. In each row, x- and y-axes shows the nucleotide number in GI-VII-6 sequence and coverage of short reads, respectively. ORFs are indicated at the bottom of each row by bold arrows.
