# Supplementary material for: Extensive amplification of GI-VII-6, a multidrug resistance genomic island of Salmonella enterica serovar Typhimurium, increases resistance to extended-spectrum cephalosporins
Source: Front Microbiol. 2015 Feb 10;6:78. doi: 10.3389/fmicb.2015.00078 (PMC4322709; doi:10.3389/fmicb.2015.00078)
Supplement: Supplementary file 6 [file Image2.PDF]

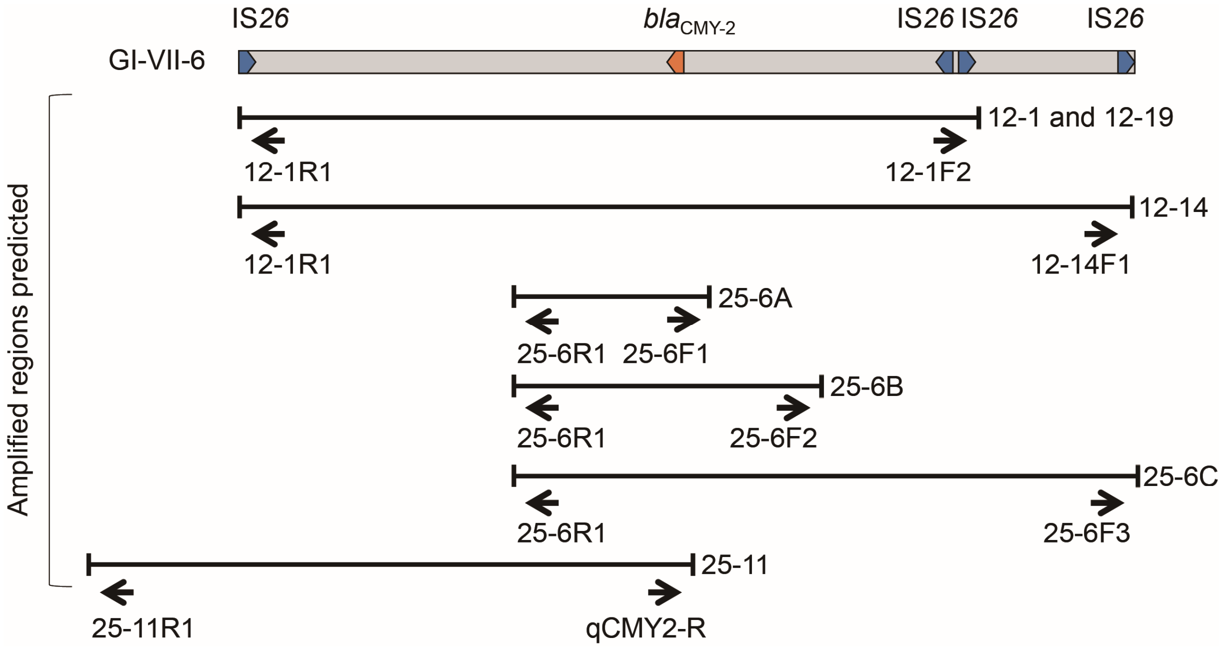

FIGURE S2. Schematic view indicating the location of primers for junction PCR. Six horizontal lines under the GI-VII-6 indicate the location of each amplified region. Position of each primer is indicated as an arrow under the horizontal lines. Name of each primer is indicated under the arrows. Primers used with each strain were as follows: strains 12-1 and 12-19, a primer pair 12-1R1 and 12-1F2; strain 12-14, a primer pair 12-1R1 and 12-14F1; strain 25-6, primer pairs 25-6R1 and either one of 25-6F1, 25-6F2, or 25-6F3; strain 25-11, a primer pair qCMY-R and 25-11R1. One of each amplified region from strains 12-1, 12-14, 12-19, and 25-11, and three different amplified regions (A, B, and C) from strain 25-6 were observed, and all the junction PCR were successful. Each PCR product was sequenced to determine the structure of the junction region.
