# Supplementary material for: Extensive amplification of GI-VII-6, a multidrug resistance genomic island of Salmonella enterica serovar Typhimurium, increases resistance to extended-spectrum cephalosporins
Source: Front Microbiol. 2015 Feb 10;6:78. doi: 10.3389/fmicb.2015.00078 (PMC4322709; doi:10.3389/fmicb.2015.00078)
Supplement: Supplementary file 7 [file Image3.PDF]

Left junction of 12-1, 12-14, and 12-19

```
IS26 (3' end)      TCATCGCTAACTTTGCAACAGTGCC
Junction           TCATCGCTAACTTTGCAACAGTGCCCTTAGAAACGTAGATCGGTGAGAGA
(983918-983967)
```

Right junction of 12-1 and 12-19

```
IS26 (5' end)                               GGCACTGTTGCAAATAGTCGGTGGT
Junction           CTTTCAGCGCTGATATCCGCTGGACGGCACTGTTGCAAATAGTCGGTGGT
(1082579-1082628)
```

Right junction of 12-14

```
IS26 (5' end)                               GGCACTGTTGCAAATAGTCGGTGGT
Junction           GAGCTTCGCGCCCAGTTTGTTCAGTGGCACTGTTGCAAATAGTCGGTGGT
(1107400-1107449)
```

Left junction in strain 25-6

```
IS26 (3' end)      TCATCGCTAACTTTGCAACAGTGCC
Junction           TCATCGCTAACTTTGCAACAGTGCCCGAGAGATTGCCCAACCAGCAGGGA
GI-VII-6           CGATTCAGCGATTCTCACGAAAGGACGAGAGATTGCCCAACCAGCAGGGA
(1021027-1021076)
```

Right junctions in strain 25-6

```
IS26 (5'end)                                GGCACTGTTGCAAATAGTCGGTGGT
Junction A         CGGCCCATTTTGGGCACTTTATGGAGGCACTGTTGCAAATAGTCGGTGGT
GI-VII-6           CGGCCCATTTTGGGCACTTTATGGAGGTTTATTCGAGGAGATGATAAGAG
(1048447-1048496)
IS26 (5' end)                               GGCACTGTTGCAAATAGTCGGTGGT
Junction B         CAGTCGCGTCGTATAAACGCCAAAAGGCACTGTTGCAAATAGTCGGTGGT
GI-VII-6           CAGTCGCGTCGTATAAACGCCAAAAGGCAATTCGCCACCTTCCCGGAACT
(1063350-1063399)
IS26 (5' end)                               GGCACTGTTGCAAATAGTCGGTGGT
Junction C         GAGCTTCGCGCCCAGTTTGTTCAGTGGCACTGTTGCAAATAGTCGGTGGT
(1107400-1107449)
```

Left junction in strain 25-11

```
IS1 (5' end)       AAATCAGTAAGTTGGCAGCATCACC
Junction           AAATCAGTAAGTTGGCAGCATCACCTGTTTTCTCTGGAATTTATTCTTAT
Chromosome         CCCGTGATTCAGGCAGAAGTGCTGCTGTTTTCTCTGGAATTTATTCTTAT
(962935-962984)
```

Right junction in strain 25-11

```
IS1 (3' end)                                GGTAATGACTCCAACTTATTGATAG
Junction           TTTTCAGAAAGCAAGGTAGTATTTGGGTAATGACTCCAACTTATTGATAG
GI-VII-6           TTTTCAGAAAGCAAGGTAGTATTTGTTACAAAGAAAAAGTAGTCGTATTC
(1042393-1042442)
```

FIGURE S3. Alignment of nucleotide sequences of the junctions of the amplified regions, insertion sequences, and GI-VII-6. Nucleotide position in GI-VII-6 (DDBJ accession number AP014565) is shown in parentheses. The existence of IS*26* was confirmed in the junction regions of strains 12-1, 12-14, and 12-19. IS*26* was also existed in the junctions of the three different amplified regions (A, B, and C) observed in strain 25-6. IS*1* was existed in the junction in strain 25-11.
